# Technologies and opportunities to improve sustainability of swine production systems

**DOI:** 10.1093/jas/skaf427

**Published:** 2025-12-12

**Authors:** Laura Greiner

**Affiliations:** Department of Animal Science, Iowa State University, Ames, IA 50011, USA

Over the past two decades, the world has had increasing discussions on environmental sustainability. Agriculture provides basic needs of humans. As the world faces current and newly evolving challenges, we are reminded that agriculture must do its part in conserving nutrients to feed a growing population with a shrinking agricultural land mass.

Depending on the animal species, ration, and management, between 5% and 45% of the nitrogen (N) in protein fed to animals is retained by the animal or incorporated into the salable product (milk, eggs, meat) (Oenema, 2006; Groenestein et al., 2019), while the remaining 55–95% is excreted in feces and urine. Retention rates of phosphorus and potassium are similar (Spears et al., 2003; Kojima et al., 2005; Vitti and Kebreab, 2010; Kebreab et al., 2012). Over the last 50 years, the U.S. (United States) swine industry has seen a reduction in the carbon footprint per kg of carcass by 35% (3.8 kg/CO_2e_ to 2.5 kg/CO_2e_; Boyd et al., 2012). This reduction in the carbon footprint has been due to the genetic selection for leaner, faster growing, and more efficient animals, along with advances in animal housing, nutrition, and health. Additionally, production practices, including manure management, in addition to the aforementioned factors, have resulted in the use of 75.9% less land, 25.1% less water, 7% less energy, and 7.7% lower carbon emissions per kg pork produced during that same time (U.S. Pork Industry 2021 Sustainability Report, 2022).

As the efficiency of size has moved through agriculture, swine production facilities have become larger relative to the land mass surrounding the facilities (MacDonald and McBride, 2009). With the larger facilities, changing feedstuffs and external farm resource use, the agricultural industry has seen distancing between livestock and crop agriculture, resulting in an atypical nutrient cycle and a complicated evaluation of nutrient utilization efficiency across the entire nutrient lifecycle (Garrett et al., 2020). To best determine swine agriculture sustainability within the global environment, a more complete lifecycle assessment (LCA) that acknowledges the boundary, scope, and circular nature of the nutrient cycle needs to be developed. This was further supported by the Food and Agriculture Organization of the United Nations (FAO) when it released a publication that provides guidelines for a complete evaluation of nutrient flow for livestock production (FAO, 2018).

A complete evaluation of nutrient flow goes beyond the four categories of nutrient flow: pigs>manure>soil>feed crops. The evaluation should in fact be circular and encompass factors of production that have direct influence on the four above mentioned categories, including genetics, regionalization, technology, etc. For example, while evaluation of the nutrient composition of manure is valuable, calculations surrounding nutrient volatilization or loss during the process of manure storage, treatment, and utilization impact relevant metrics at a systems level. Despite the importance of nutrient recycling, current estimates suggest that only half of the nitrogen in animal manure is captured, placed in storage, and land applied (Oenema and Tamminga, 2005). The precise amount of nitrogen lost (mostly volatilized) varies widely based on the design of the manure management system (Sommer et al., 2019). For example, deep pits volatilize 15–30% of the N received, compared to 50–70% for lagoon systems. Holding more N and using it to support crop production (corn or grain) improves system-level N use efficiency. Creating that circularity at a system level such as a farm (or farms, depending on the integration of crop and livestock production in the area) can be complex based upon regional feed ingredient availability. Furthermore, there are multiple boundary level approaches which could be considered to undertake the LCA, including assessment at field-scale, whole-farm, or regional/watershed levels (Sharara et al., 2022). Each of these models has challenges in fully accounting for nutrient utilization and efficiency. Therefore, there is a need to determine the “best-fit” model to develop a formal system to help producers, agronomists, nutritionists, environmentalists, and others to best determine where gaps and opportunities exist.

While much progress has been made in estimating nitrogen utilization and loss around feed, manure, soil, and crops independently, there is much work yet to be done in defining other nutrients such as sulfur and carbon, as well as creating the circulatory associated with nutrient recycling. The purpose of the reviews included in this special series is to provide focus on the state of knowledge information associated with these key areas of the nutrient cycle and to identify gaps in knowledge and opportunities.
